# Cholesterol and CDON Regulate Sonic Hedgehog Release from Pancreatic Cancer Cells

**DOI:** 10.1089/pancan.2021.0002

**Published:** 2021-06-01

**Authors:** Jennifer I. Alexander, Esteban Martinez, Alberto Vargas, Daniel Zinshteyn, Valerie Sodi, Denise C. Connolly, Tiffiney R. Hartman, Alana M. O'Reilly

**Affiliations:** ^1^ Molecular Therapeutics, Fox Chase Cancer Center, Philadelphia, Pennsylvania, USA.; ^2^ Roberts Individualized Medical Genetics Center and the Division of Human Genetics, Children's Hospital of Philadelphia, Philadelphia, Pennsylvania, USA.

**Keywords:** CDON, cholesterol, pancreatic cancer, Sonic Hedgehog

## Abstract

**Background:** Sonic Hedgehog (Shh) is a tightly regulated membrane-associated morphogen and a known driver of tumorigenesis in pancreatic ductal adenocarcinoma (PDAC). After processing, Shh remains at the plasma membrane of Shh producing cells, thereby limiting its distribution and signal strength. In PDAC, the release of Shh from tumor cells is necessary to promote a tumor-permissive microenvironment. Mechanisms regulating Shh sequestration and/or release from tumor cells to signal distant stromal cells are not well known. Previously, our laboratory demonstrated that the Drosophila transmembrane protein Boi, sequesters Hh at the membrane of Hh-producing cells. In response to dietary cholesterol or in the absence of boi, Hh is constitutively released to promote proliferation in distant cells. In this study, we investigated the conservation of this mechanism in mammals by exploring the role of the human boi homolog, CDON, in PDAC.

**Methods:** Using PDAC cell-lines BxPC-3, Capan-2, and MIA PaCa-2, along with normal pancreatic epithelial cells (PDEC), we investigated Shh expression via Immunoblot and real-time, quantitative polymerase chain reaction in addition to Shh release via enzyme-linked immunoassay following cholesterol treatment and/or transfection with either RNA interference to reduce CDON expression or with human *CDON* to increase expression.

**Results:** Consistent with our Boi model, CDON suppresses Shh release, which is alleviated in response to dietary cholesterol. However, over-expressing CDON suppresses cholesterol-mediated Shh release in some PDAC contexts, which may be relative to the mutational burden of the cells.

**Conclusion:** Identifying mechanisms that either sequester or stimulate Shh release from the tumor cell membrane may provide new avenues to reduce signaling between the tumor and its surrounding environment, which may restrain tumor development.

## Introduction

Aberrant Sonic Hedgehog (Shh) expression occurs in early benign pancreatic precursor lesions, contributing to progressive changes in pancreatic stroma.^1^ Shh-mediated stromal remodeling contributes to poor response rates in patients undergoing standard-of-care therapies,^2,3^ suggesting that targeting mechanisms that restrict Shh release might prevent progression to pancreatic ductal adenocarcinoma (PDAC) by reducing the stromal therapeutic barrier. Previously, our laboratory defined a multistep Hedgehog (Hh)-dependent molecular pathway in which the transmembrane protein Boi interprets nutrient signals to control epithelial stem cell proliferation in *Drosophila* ovaries.^4^ Boi binds to Hh in apical somatic cells through an extracellular fibronectin III repeat, sequestering Hh at the surface of Boi-expressing cells. Strikingly, Hh is sequestered under poor nutrient conditions and robustly released rapidly upon feeding. We identified cholesterol as a key regulator of Hh sequestration and release,^4,5^ with cholesterol feeding activating an inside-out signaling response to trigger Hh release from apical cells followed by induction of proliferation of epithelial follicle stem cells located 8–10 cell diameters to the posterior.

Cholesterol is required for effective signal transduction of the Hh pathway.^6–8^ PDAC retrospective outcome studies suggest that reducing cholesterol, through statin treatment, improves patient survival in early stages of disease.^9,10^ In addition, statin treatment significantly delayed PDAC progression in a genetic mouse model of pancreatic cancer.^11,12^ Moreover, meta-analysis of 26 studies found significantly decreased pancreatic cancer risk with statin use.^13^ It may be that statin use contributes to suppression of Shh release and/or signal transmission, thereby preventing Shh-mediated stromal remodeling. In this study, we investigate conservation of the fly mechanism in human PDAC cells. We demonstrate that cholesterol is sufficient to trigger Shh release, suggesting a potential role for this pathway in PDAC development and progression. The mammalian Boi family member, CDON (cell adhesion molecule-related, down-regulated by oncogenes), suppresses Shh release in PDAC cells, and may autonomously regulate PDAC cell proliferation.^14^ These results support the conservation of key aspects of cholesterol-regulated Shh release in PDAC. Taken together, this study adds to our understanding of mechanisms of Shh regulation with potential implications for leveraging Shh sequestration and release to improve therapeutic interventions for pancreatic cancer patients.

## Materials and Methods

### Human pancreatic cancer cell lines

Authenticated human pancreatic ductal epithelial cells (PDEC) were cultured in Dulbecco's modified Eagle medium (DMEM) supplemented with M3 BaseF media, 1 g/L NaHCO_3_, pyruvate, 5% fetal bovine serum (FBS), 1% penicillin/streptomycin (1% Pen/Strep). Culture media for authenticated PDAC cells: BxPC3: RPMI-1640 containing 2 g/L NaHCO_3_, 10% FBS, and 1% Pen/Strep, Capan-2: McCoy's 5A modified with 2.2 g/L NaHCO_3_, 10% FBS, and 1% Pen/Strep, MIAPaCa-2: DMEM containing 10% FBS, 2.5% Horse Serum, and 1% Pen/Strep.

### Cell starvation and cholesterol treatment

Cells were plated onto six-well plates at confluency overnight. Cells were rinsed twice with phosphate buffered saline (PBS) before incubating overnight with 2 mL/well of Hank's buffer saline solution (HBSS) or McCoy's media. HBSS was replaced with fresh HBSS or McCoy's media containing 10% FBS, 10% lipid depleted serum, or 4 μL/mL of 250 × Cholesterol Lipid Concentrate (contains fatty acids and synthetic cholesterol) for 6 h before conditioned media was collected.

### Western blot analysis

Confluent cells were lysed in RIPA buffer, homogenized, and diluted in 2 × loading buffer before boiling (10 min), and separated in 4–20% sodium dodecyl sulphate–polyacrylamide gel electrophoresis. Gels were transferred to polyvinylidene difluoride membranes and blocked for 1 h at room temperature (RT) in 5% milk in 0.1% Tris Buffered Saline and Tween, incubated overnight in primary antibody and 2 h in secondary antibody before developing. Primary antibodies: rabbit polyclonal anti-CDON (Thermo Fisher Scientific), rabbit polyclonal anti-Shh (Abcam), rabbit anti-beta-actin (Abcam), and mouse anti-alpha-Tubulin (Sigma-Aldrich). Secondary antibodies: anti-rabbit or anti-mouse IgG-Horseradish Peroxidase (Cell Signaling Technology).

### Quantitative reverse transcriptase polymerase chain reaction

Total RNA from PDEC and PDAC cells was extracted, following Qiagen RNeasy Mini Kit manufacturer's instructions. RNA was reverse transcribed with a mixture of anchored oligo-dT and random decamers (Ambion, Life Technologies). Taqman assays were used in combination with Taqman Universal Master Mix (Thermo Fisher Scientific) and run on a 7900 HT sequence detection system (Applied Biosystems). Cycling conditions: 95°C (15 min) followed by 40 cycles of denaturation at 95°C (15 sec) and annealing and extension at 60°C (60 sec). Messenger RNA (mRNA) copy number was calculated from serially diluted standard curves generated from complementary DNA templates. Reactions were performed with two technical replicates, with average quantity normalized to housekeeping gene ± standard error. Primer sequences:

Shh Hs00179843_m1
Dispatched-1 (DISP) Forward: TGCAATGTAGATAATTCCAGGATC
Reverse: TCAGAATGGCGATGTAGTTTCC
SCUBE2 Forward: CCAGAGAGACTGCATCTTGAC
Reverse: TGCATCTTGTACTGTGGATGG
Brother of CDON (BOC) Hs00264408_m1
CDON Forward: GTTAACTGCCGAAATTGTCGAAA
Reverse: TGCTACCACAGGGACCACAG

### Lentiviral production and transduction

pLKO1.sh*CDON*.GFP: *shRNA* guides targeting human *CDON* were cloned into the AgeI and EcoRI sites of the pLKO1.GFP vector. *CDON* targets were acquired from the TRC shRNA library and knockdowns were validated through western blot. The top two knockdown candidates were kept for subsequent studies (shRNA CDON #1: TRCN0000073396 and shRNA CDON #2 TRCN0000073393). 293T cells treated with X-Treme Gene 9 transfection reagent (XTG9-RO; Sigma-Aldrich) were transfected with sh*CDON* plasmid along with pMD and pPAK packaging plasmids to produce high-titer lentivirus. Viral supernatants were frozen (−80°C) until further use. 5 × 10^5^ PDAC cells were subjected to spin transduction with recombinant pLKO1.GFP-expressing lentivirus targeting human *CDON* (multiplicity of infection 0.4–0.6). Ninety-six hours after transduction, cells were sorted based on GFP expression by fluorescence-activated cell sorting (FACS) using the FACS Aria II (Becton, Dickinson, and Company) and plated in 10 cm dishes.

### Immunofluorescence

Samples were prepared for immunofluorescence as published previously.^4,5^ Samples were fixed with 4% paraformaldehyde and permeabilized before 1-h blocking using 1 × PBS containing 10% horse serum. Afterward, samples were incubated overnight at 4°C with primary antibodies: polyclonal rabbit anti-Shh (1:100; Abcam) and sheep anti-CDON (1:100; R&D Systems). After three 5 min PBS-Tween20 (0.05%) rinses, secondary antibodies were incubated for 1 h (1:200) followed by Draq-5 (1:1,000; Thermo Fisher Scientific) stain. Samples were rinsed before mounted using Vectashield (Vector Laboratories). Images were collected on an upright microscope (DM5000 B; Leica) coupled to a confocal laser scanner (TCS SP5; Leica). LAS AF SP5 software (Leica) was used for data acquisition and analysis.

### ELISA assay

Shh Human ELISA Kit (ab100639; Abcam) was used as instructed by the manufacturer. In brief, conditioned media was collected from PDAC cells cultured in HBSS or cholesterol-supplemented HBSS. Conditioned media was concentrated using Pierce Protein Concentrator PES 10K MWCO (Thermo Fisher Scientific) and centrifuged at 4000 *g* (10 min at 4°C). Samples and standards were incubated overnight on precoated 96-well plates provided in the kit. After three washes, wells were incubated for 1 h (RT) with detection antibody. Wells were washed thrice before incubating with streptavidin-HRP (45 min at RT), then washed 3 × and incubated in substrate solution (30 min) before stop solution was added. Optical density readings at 450 and 570 nm were collected using the Synergy H1 plate reader (BioTek). Shh concentration (pg/mL) was normalized to the amount of protein in each well, with the modal value referred to as one arbitrary unit.

### Statistical analysis

Each experiment comprised two biological experiments, each with three technical replicates (unless stated otherwise). GraphPad Prism 8.0 software was used for statistical analysis: Comparison between two groups: two-tailed unpaired Student's *t*-test; Experiments with three or more conditions, one-way analysis of variance with multiple comparisons set to control cells was conducted (unless noted otherwise). Significance is defined as *p < 0.05; **p < 0.01; ***p < 0.001; ****p < 0.0001.

## Results

### Cholesterol stimulates Shh release in human PDAC cells

**FIG. 1.**
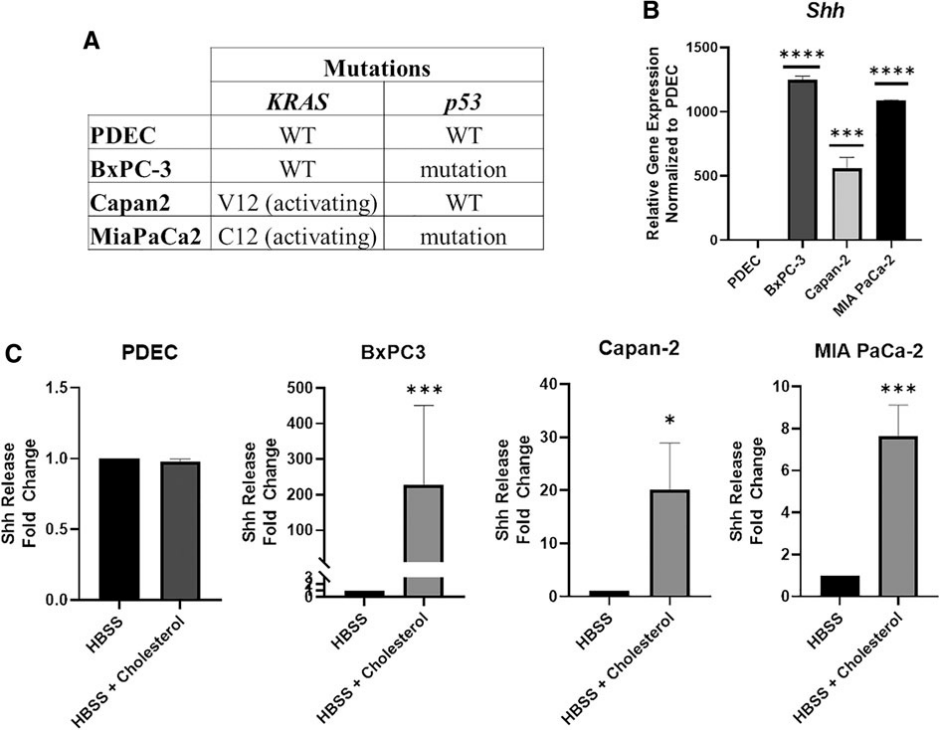
Cholesterol stimulates Shh release in human PDAC cells. **(A)** Table highlights the primary driver mutations in each PDAC cell line along with tumor differentiation status where PDEC and Capan-2 are the most differentiated, BxPC-3 is moderately differentiated, and MIA PaCa-2 are poorly differentiated. **(B)** RT-qPCR data measuring relative gene expression of *Shh* in PDAC cells normalized to PDEC control cells. **(C)** ELISA results measuring fold-change differences in the amount of Shh released in PDAC cells stimulated with HBSS + cholesterol relative to HBSS alone. Statistical significance denoted by asterisks were *p < 0.05, ***p < 0.001, and ****p < 0.0001. Lack of asterisks indicates data were not significant. ELISA; HBSS, Hank's buffer saline solution; PDAC, pancreatic ductal adenocarcinoma; PDEC, pancreatic ductal epithelial cells; RT-qPCR, quantitative reverse transcriptase polymerase chain reaction; Shh, Sonic Hedgehog; WT, wild-type.

Our first goal was to determine whether exogenous cholesterol could stimulate Shh release in pancreatic cells. To test this, we used immortalized, nontransformed human PDEC and patient-derived PDAC cell lines bearing common PDAC driver mutations (*TP53* and *KRAS*) with varying tumor differentiation status (Fig. 1A).^15^ BxPC-3 PDAC cells were derived from tumor tissue with differentiation phenotypes ranging from moderate to poor, and bear mutations in *TP53* but not *KRAS.*^16^ Capan-2 PDAC cells were initially derived from a well-differentiated tumor and bear *KRAS*, but no *TP53* mutation.^16^ MIA PaCa-2 PDAC cells were derived from a poorly differentiated tumor and bear mutations in both *TP53* and *KRAS.*^16–18^ All three cell lines display high Shh expression (BxPC-3 *p* < 0.0001, Capan-2 *p* = 0.0006, MIA PaCa-2 *p* < 0.0001) relative to nontransformed PDEC (Fig. 1B), consistent with previous literature reporting elevated Shh signaling in PDAC.^1,3^ These cell lines model the PDAC continuum, providing a competent system for the investigation of cholesterol-mediated regulation of Shh in various PDAC stages of development.

We analyzed cholesterol-mediated Shh release in PDEC and PDAC cells, before and after stimulation with cholesterol, through ELISA. Cholesterol failed to stimulate Shh release from PDEC (*p* = 0.1000), in contrast to all three PDAC cell lines, which significantly released Shh in response to cholesterol (BxPC-3 *p* = 0.0003, Capan-2 *p* = 0.0286 and MIA PaCa-2 *p* = 0.0002; Fig. 1C). Previous study demonstrated that a Shh precursor protein is cleaved to generate active Shh (Shh-C). If cholesterol-stimulated release promotes increased Shh activity, then levels of cleaved Shh (Shh-C) should increase upon cholesterol treatment. Consistent with this idea, levels of cleaved Shh (Shh-C) in BxPC-3 and Capan-2 cells increased under cholesterol treatment (Supplementary Fig. S1A). Levels of uncleaved inactive full-length Shh (Shh-FL) were unchanged (Supplementary Fig. S1A). In Capan-2 cells, cholesterol alone or full serum, which contains high levels of cholesterol, both induced Shh release (*p* = 0.0155) and (*p* = 0.0005), respectively (Supplementary Fig. S1B). In contrast, treatment with lipid-depleted media failed to stimulate Shh release, with a slight but significant reduction relative to no treatment (*p* = 0.0433; Supplementary Fig. S1C). These results support the model that cholesterol triggers Shh release into the PDAC microenvironment.

### CDON is upregulated in human PDAC cells

**FIG. 2.**
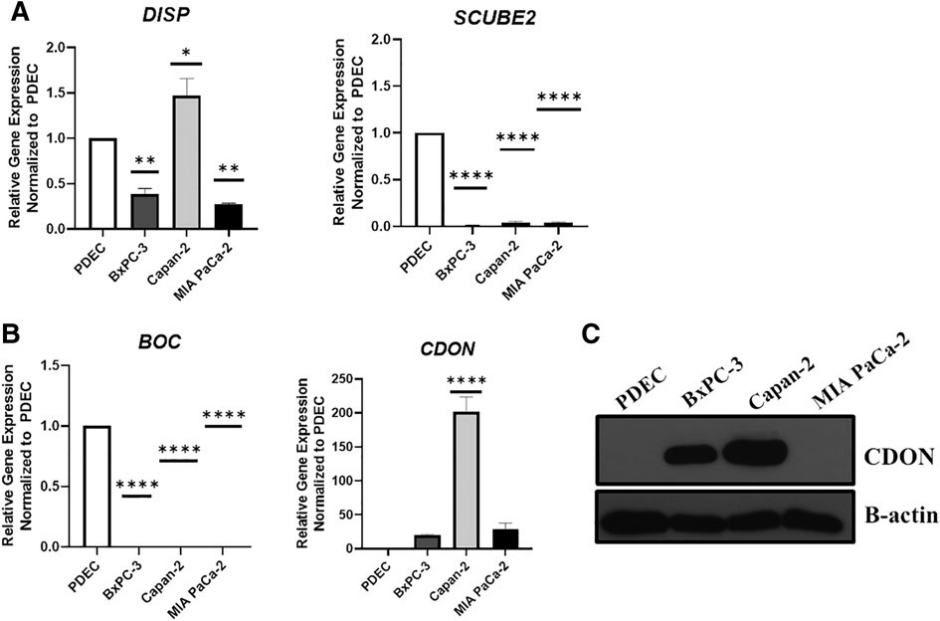
CDON is upregulated in human PDAC cells. **(A, B)** RT-qPCR data measuring relative gene expression of *DISP, SCUBE2, BOC,* and *CDON* in PDAC cells relative to PDEC. **(C)** Western blot image of CDON expression in PDEC and PDAC (Capan-2, BxPC-3, and MIA PaCa-2) cells. Of note, at this exposure, endogenous CDON protein is not detected in MIA PaCa-2 cells. B-actin served as the loading control. Statistical significance denoted by asterisks were *p < 0.05, **p < 0.01, and ****p < 0.0001. Lack of asterisks indicates data were not significant.

Multiple proteins have been identified that can regulate Shh release. DISP and Scube2 can function in a co-dependent relay mechanism to promote Shh release.^19^ *DISP* levels were two- and threefold lower than PDEC in BxPC-3 and MIA PaCa-2, respectively (*p* = 0.0059 and 0.0021; Fig. 2A), despite robust cholesterol-induced Shh release (Fig. 1). Capan-2 cells expressed slightly elevated *DISP* (*p* = 0.0244), but *Scube 2* levels were exceptionally low in all three PDAC cells lines relative to PDEC (*p* < 0.0001), suggesting that another mechanism must mediate Shh release in a PDAC setting.

We next measured expression levels of the Boi homologs BOC and CDON, which bind to Shh and regulate Shh signaling in mice.^20–22^ *BOC* gene expression was absent in PDAC cells relative to controls (*p* < 0.0001), whereas *CDON* was significantly elevated in Capan-2 cells (*p* < 0.0001) and detected in BxPC-3 and MIA PaCa-2 cells, although expression was not statistically different from PDEC controls (*p* = 0.5808 and *p* = 0.3111, respectively; Fig. 2B). Compared with our control PDEC, a western blot analysis revealed increased CDON expression in BXPC-3 and Capan-2 cells (Fig. 2C), suggesting differential post-transcriptional regulation to increase CDON levels in PDAC versus untransformed cells.

### CDON knockdown in Capan-2 and MIA PaCa-2 increases Shh release

**FIG. 3.**
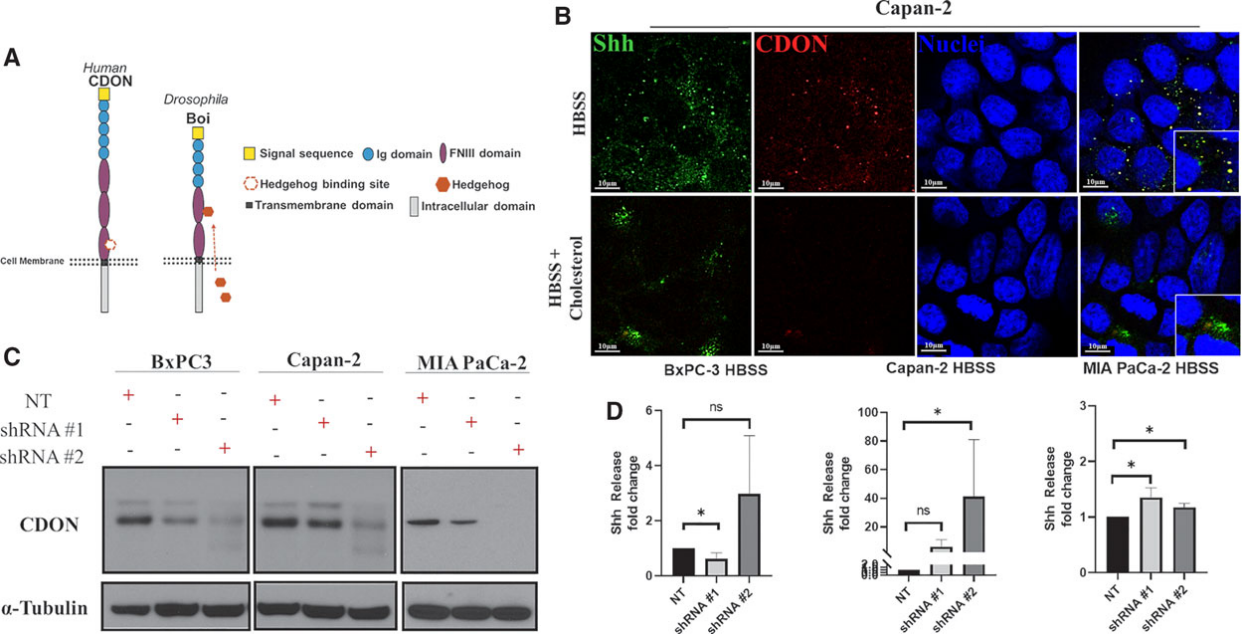
CDON knockdown in Capan-2 and MIA PaCa-2 increase Shh release. **(A)** Schematic of the structural domains of the human and fly homologs CDON and *Boi*, respectively. Shh binding site on the FNIII domain of CDON and *Boi* is depicted in orange. **(B)** Immunofluorescence images of Shh (green) and *CDON* (red) colocalizing (yellow) in Capan-2 cells (nuclei in blue) treated with HBSS alone. Insets are magnifications of merged images to highlight reduced colocalization (absence of yellow) in Capan-2 cells after HBSS + cholesterol. Scale bar 10 μm. **(C)** Representative western blot image of *CDON* relative expression in PDAC cells transfected with either NT shRNA or 2 *CDON* targeting shRNA (shRNA #1 or shRNA #2). α-Tubulin served as the loading control. **(D)** Fold-change differences in the amount of Shh released from PDAC cells transfected with NT versus *CDON* targeting shRNAs under starved conditions relative to PDEC controls. Statistical significance denoted by asterisks were *p < 0.05. NS indicates data were not significant. FNIII domain, fibronectin type III domain; NT, nontargeting.

CDON shares significant structural similarity with its *Drosophila* homolog Boi, including a conserved fibronectin type III domain Hh binding site (Fig. 3A).^23–26^ If CDON sequesters Shh to limit its release upon cholesterol stimulation, we expected to see enhanced Shh release in (1) cells treated with cholesterol and (2) cells with reduced *CDON* expression regardless of serum cholesterol levels. Imaging analysis revealed colocalization of Shh and CDON under HBSS treatment, which occurred less under cholesterol (Fig. 3B), consistent with reduced CDON-mediated Shh sequestration upon cholesterol treatment. *CDON* levels were reduced by expressing two different shRNA constructs (shRNA #1 and shRNA #2). *CDON* protein knockdown relative to nontargeting (NT) control was confirmed by immunoblotting (Fig. 3C). Shh released from CDON knockdown cells was then measured relative to NT control (Fig. 3D). MIA PaCa-2 and Capan-2 cells exhibited enhanced Shh release (shRNA #1 *p* = 0.0286) and (shRNA #2 *p* = 0.0434), respectively. In contrast, BxPC-3 cells had a distinct response to reduced CDON expression, whereby weak CDON knockdown led to a statistically significant reduction in Shh release (shRNA #1 *p* = 0.0387), whereas strong knockdown did not (shRNA #2 *p* = 0.9532). All three cell lines bear many mutational differences that might explain the distinct response. For example, Capan-2 and MIA PaCa2 share *KRAS* mutation, whereas BxPC3 cells have wild-type *KRAS*. A previous study suggests possible mechanistic links between Kras and CDON^27^ with implications for predicting outcomes of CDON expression on Shh release in PDAC.

### Overexpressing CDON suppresses Shh release

**FIG. 4.**
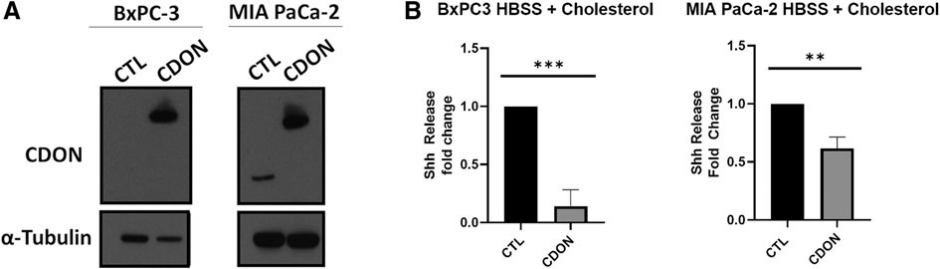
Overexpressing CDON suppresses Shh release. **(A)** Representative western blot image of CDON relative expression in PDAC cells transfected with plasmids expressing *CDON* versus GFP CTL to yield cells that overexpressed *CDON*. Note, at this exposure, endogenous CDON protein is not detected. α-Tubulin served as the loading control. **(B)** Fold-change differences in the amount of Shh released from PDAC cells transfected with CTL versus *CDON* overexpressing plasmid treated with cholesterol relative to CTL. Statistical significance denoted by asterisks were **p < 0.01 and ***p < 0.001. CTL, control.

If CDON functions to limit Shh secretion in PDAC cells, we expect overexpressing CDON to suppress the effects of cholesterol stimulation on Shh release. To test this, we stably expressed GFP and/or CDON.GFP in PDAC cells through lentiviruses that (1) express GFP alone (control) or (2) co-express GFP and *CDON*. After transduction, lentivirus infection was confirmed by FACS, with all three cell lines exhibiting GFP expression. GFP-sorted cells were plated and CDON overexpression was confirmed by immunoblotting before cells were expanded to assess effects on Shh release (Fig. 4A). Capan-2 cells, which exhibit the highest levels of endogenous CDON mRNA and protein, were not viable after transduction, suggesting that excess CDON negatively impacts survival. In contrast, BxPC-3 and MIA PaCa-2 cells significantly overexpressed CDON relative to controls and retained this overexpression for several passages after sorting (Fig. 4A). Upon cholesterol treatment, BxPC-3 and MIA PaCa-2 cells overexpressing CDON released five- and twofold less Shh relative to control cells (*p* = 0.0009 and *p* = 0.0019), respectively, (Fig. 4B), indicating that CDON overexpression hinders cholesterol-induced Shh release, likely through sequestration.

## Discussion

In this study, we report a mechanistic conservation between *Drosophila boi* and human *CDON* in regulating Shh release in PDAC cells (Capan-2 and MIA PaCa-2). In both fruit fly and human systems, loss of *boi*/*CDON* results in increased release of Hh or Shh in the absence of cholesterol (Fig. 3D).^4,5^ Conversely, CDON overexpression can reduce the amount of Shh released under cholesterol stimulation, suggesting that CDON may limit Shh release into the tumor microenvironment. BxPC-3 exhibited little change upon altered CDON expression, suggesting that the impact of CDON on Shh release may be dependent on mutational status in PDAC.

In the fruit fly, Boi is required for both sequestering and releasing Hh in response to cholesterol. In contrast, our results suggest that the role of CDON in PDAC is to limit Shh release, as overexpression of CDON reduced Shh release rather than synergizing with cholesterol stimulation to promote it. In fruit flies, cholesterol activates S6 Kinase, which phosphorylates Boi to trigger release of sequestered Hh. Interestingly, CDON lacks an S6K target site, which may explain why CDON suppresses, but does not facilitate Shh release. Ultimately, this indicates that Shh sequestration and release are distinctly regulated processes in human cells. We also considered DISP and Scube2 as potential regulators of cholesterol-mediated Shh release, finding limited DISP expression PDAC cells, but no Scube2 expression. The absence of Scube2 excludes regulation of Shh release by the previously described DISP-Scube2 mechanism, but it remains possible that low expression of DISP may contribute to Shh release through a novel mechanism. Cholesterol influences multiple aspects of Shh pathway regulation, including direct binding to Shh ligand and Smoothened to enhance Shh signaling,^7,8^ which might contribute to regulation of Shh levels in PDAC.

A major challenge in detecting PDAC is the lack of early disease markers. As a result, treatment is typically administered when the tumors are already advanced in stage. Here we show that CDON is upregulated in human PDAC cells to varying degrees, consistent with reported Shh expression in a mouse PDAC model.^1,28^ CDON expression may be useful for identifying tumors that are sensitive to Shh release or for assessing the differentiation status of tumors and their associated stroma. Capan-2 cells are derived from well-differentiated tumors, express the highest amount of CDON in comparison with nontransformed PDEC, and exhibit the highest degree of sensitivity to changes in CDON expression. MIA PaCa-2 cells express very low amounts of CDON at the protein level and are derived from a poorly differentiated tumor. Thus, CDON expression levels may provide opportunities to determine the differentiation status of PDAC tumors and predict the impact of CDON targeting on Shh release and tumor progression. In fact, pancreatic tumors depend on Shh-mediated stromal remodeling to promote tumor development. One study suggests that membrane tethering of Shh increases its signaling potency to elicit pro-tumor stromal remodeling.^29^ This extensive stromal response contributes to the poor response rate of patients to standard-of-care therapies.^2,3^ Recent studies demonstrates that reducing cholesterol through statin use early in PDAC progression has benefits,^9,10,13^ whereas long-term statin use may increase the risk of promoting cancer onset.^30^ This complex, temporal response suggests that defining the precise contributions of bound versus released Shh in PDAC development, as well as the relationship of cholesterol to Shh signaling at various stages, will further enhance our understanding of the roles of Shh signaling in this aggressive disease.

## Conclusion

Dietary nutrients are known to be critical for the development and maintenance of healthy tissue. Although diet has been implicated in resolving or exacerbating various ailments, the signaling mechanisms by which most nutrients function remain elusive. We demonstrate that cholesterol stimulates the release of Shh from PDAC cells, a key pathway known to drive tumorigenesis. We also found that CDON, a transmembrane Shh receptor, suppresses cholesterol-mediated Shh release in some PDAC contexts. Our study supports conservation of aspects of our recently described cholesterol-regulated Hh-release pathway in *Drosophila*, including use of a CDON family member to regulate Shh availability. Ultimately, our study may be beneficial in identifying potential biomarkers/therapeutic targets in PDAC that minimize Shh availability in the tumor microenvironment.

## Supplementary Material

Supplemental data

## Abbreviations Used

1% Pen/Strep: 1% penicillin/streptomycin
BOC: brother of CDON
Boi: brother of ihog
CDON: cell adhesion molecule-related, down-regulated by oncogenes
CTL: control
DISP: dispatched-1
DMEM: Dulbecco's modified Eagle medium
FACS: fluorescence-activated cell sorting
FBS: fetal bovine serum
FCCC: Fox Chase Cancer Center
FNIII domain: fibronectin type III domain
GFP: green fluorescent protein
HBSS: Hank's buffer saline solution
Hh/Shh: Hedgehog/Sonic Hedgehog
mRNA: messenger RNA
NT: nontargeting
PBS: phosphate buffered saline
PDAC: pancreatic ductal adenocarcinoma
PDEC: pancreatic ductal epithelial cells
RT: room temperature
RT-qPCR: quantitative reverse transcriptase polymerase chain reaction
S6K: ribosomal protein S6 kinase
shRNA: short hairpin RNA
