# Supplementary material for: Cholesterol and CDON Regulate Sonic Hedgehog Release from Pancreatic Cancer Cells
Source: J Pancreat Cancer. 2021 Jun 1;7(1):39–47. doi: 10.1089/pancan.2021.0002 (PMC8252898; doi:10.1089/pancan.2021.0002)
Supplement: Supplemental data [file Supp_FigureS1.docx]

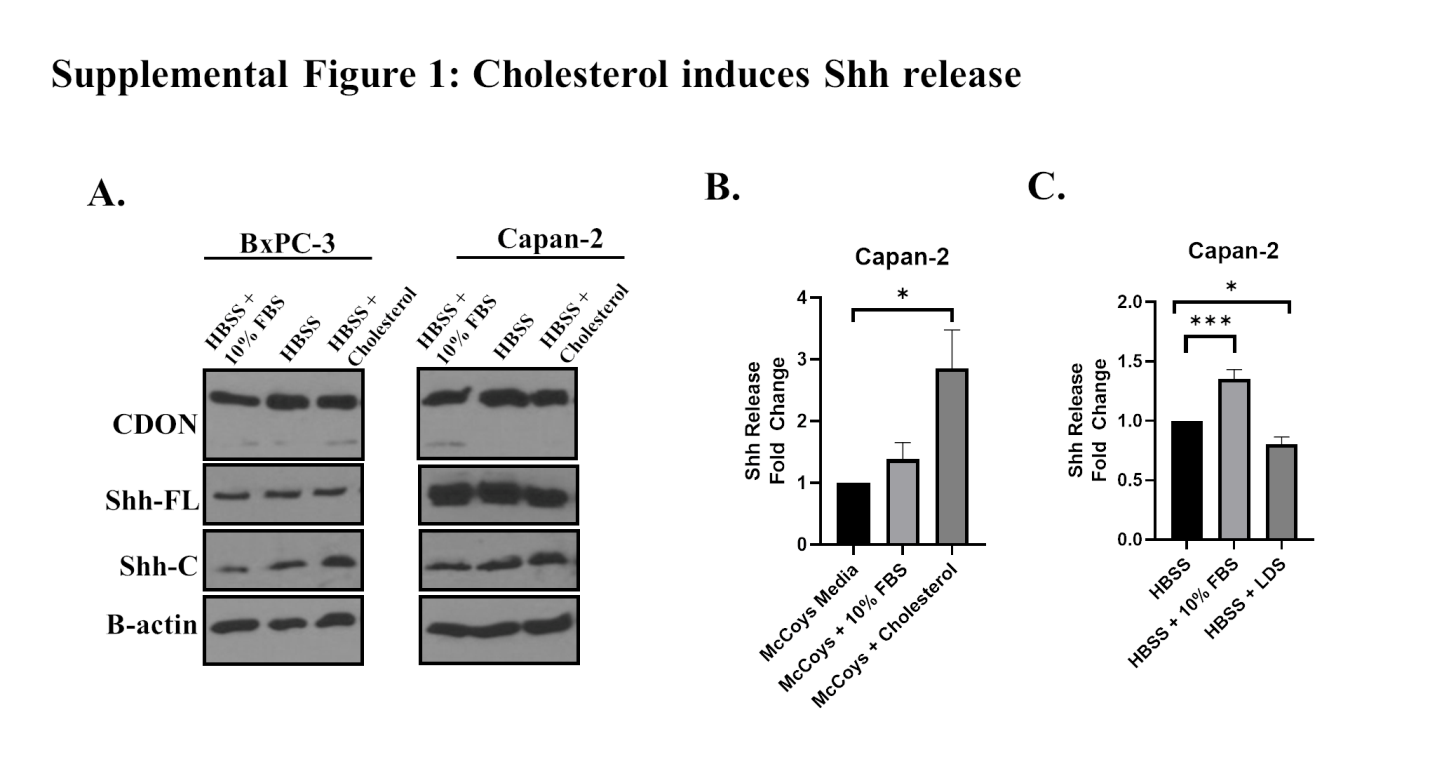


**Supplemental Figure 1: *Cholesterol induces Shh release*. A.** Western Blot image of CDON, Full Length Shh (Shh-FL), and Cleaved Shh (Shh-C) expression in BxPC-3 and Capan-2 cells cultured for 6 hours in HBSS, HBSS + 10% FBS, or HBSS + Cholesterol. В-actin served as the loading control. **B.** ELISA results measuring fold change differences in the amount of Shh released in Capan-2 cells stimulated with McCoy’s media + 10% FBS or McCoy’s media + Cholesterol relative to McCoy’s media alone. **C.** ELISA results measuring fold change differences of Shh released in Capan-2 cells treated with HBSS + 10% FBS or HBSS + Lipid depleted serum (LDS) relative to HBSS alone. Statistical significance denoted by asterisks were p<0.05 *, p<0.01 **, p<0.001 ***, and p<0.0001 ****. Lack of asterisks indicates data was not significant.
